# Effects of Caloric Restriction on Cardiac Oxidative Stress and Mitochondrial Bioenergetics: Potential Role of Cardiac Sirtuins

**DOI:** 10.1155/2013/528935

**Published:** 2013-03-18

**Authors:** Ken Shinmura

**Affiliations:** Division of Geriatric Medicine, Department of Internal Medicine, Keio University School of Medicine, 35 Shinanomachi, Shinjuku-ku, Tokyo 160-8582, Japan

## Abstract

The biology of aging has not been fully clarified, but the free radical theory of aging is one of the strongest aging theories proposed to date. The free radical theory has been expanded to the oxidative stress theory, in which mitochondria play a central role in the development of the aging process because of their critical roles in bioenergetics, oxidant production, and regulation of cell death. A decline in cardiac mitochondrial function associated with the accumulation of oxidative damage might be responsible, at least in part, for the decline in cardiac performance with age. In contrast, lifelong caloric restriction can attenuate functional decline with age, delay the onset of morbidity, and extend lifespan in various species. The effect of caloric restriction appears to be related to a reduction in cellular damage induced by reactive oxygen species. There is increasing evidence that sirtuins play an essential role in the reduction of mitochondrial oxidative stress during caloric restriction. We speculate that cardiac sirtuins attenuate the accumulation of oxidative damage associated with age by modifying specific mitochondrial proteins posttranscriptionally. Therefore, the distinct role of each sirtuin in the heart subjected to caloric restriction should be clarified to translate sirtuin biology into clinical practice.

## 1. Introduction

Aging is characterized by a progressive deterioration in physiological functions and metabolic processes, leading to an increase in morbidity and mortality. Although many theories have been proposed to explain the aging process, neither of them appears to be fully satisfactory.  Table 1 presents a summary of the major aging theories to date [1, 2]. The free radical theory of aging originally stated that free radicals generated endogenously cause oxidative modification of cellular components; the accumulation of oxidative damage with aging results in cellular dysfunction and eventually cell death [3–5]. The free radical theory has been expanded in light of the fact that reactive oxygen species (ROS) are constantly produced in cells under normal condition and cells have a higher antioxidant capacity per se [3, 5–7]. The oxidative stress theory of aging proposes that the formation of ROS including nonradical hydrogen peroxide and reactive nitrogen species, peroxynitrite, is the major generator of cellular damage and senescence. The age-related increase in oxidative damage to DNA, lipids, and proteins has been well documented [3, 5–7]. The mitochondrion is the main source and target of ROS. Mitochondria have been a central focus of the aging theory because of their critical role in bioenergetics, oxidant production, and regulation of cell death [7–12]. According to the hypothesized role of mitochondria in the aging process, organs that exhibit high rate of oxygen consumption throughout an individual's lifetime, such as the heart, brain, and kidney, may be especially prone to oxidative damage [8]. However, ROS are not just unwanted byproducts of oxidative phosphorylation in the respiratory chain; they are also highly regulated signal molecules involved in the cellular stress response.

Numerous experimental interventions designed to regulate the aging process have been attempted heretofore. To date, an established intervention that has been consistently shown to slow the rate of aging and to increase both mean and maximal lifespan in various species is lifelong caloric restriction (CR) [11, 13, 14]. The beneficial effects of lifelong CR may derive, at least in part, from a reduction of oxidative damage in organs and tissues [8, 11–15]. In contrast, although overexpression of antioxidant enzymes and antioxidant supplement diets have had some degree of success in attenuating age-associated physiological dysfunction and extending mean lifespan, they have not extended maximal lifespan [16–19]. Clinical investigations demonstrate that antioxidant treatment has either no effect or detrimental effects on health beneficial outcomes in cancer, diabetes, cardiovascular disease, and overall mortality [20, 21]. These results suggest that modest ROS production promotes longevity by inducing the innate adaptive response against oxidative stress; this mechanism may be essential for the development of overall stress resistance and lifespan extension [19, 22]. A favorable response to a low dose of poison is called hormesis. By extension, the beneficial response of mitochondria-derived ROS is named mitohormesis [22]. Since there are contradictory reports on the impact of exogenous antioxidant treatment on mitochondrial biogenesis and endogenous antioxidant defense, experiments that are more definitive are needed to address this issue.

In this paper, we discuss how cardiac mitochondrial dysfunction and oxidative stress contribute to cardiac aging and how CR regulates them.

## 2. Mitochondrial Theory of Aging

**Figure 1:**
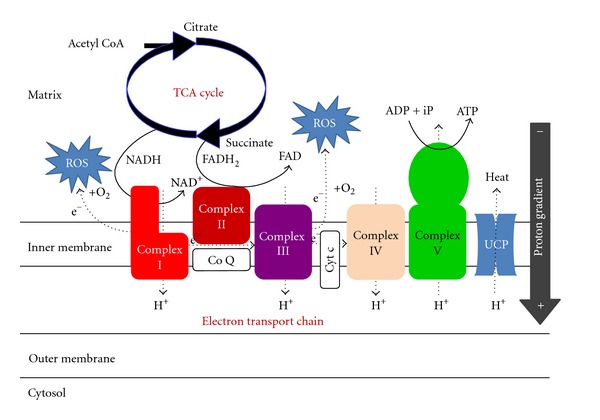
The electron transport chain (ETC) responsible for ATP and ROS production in mitochondria. ROS: reactive oxygen species, TCA: tricarboxylic acid, NADH & NAD^+^: nicotinamide adenine dinucleotide reduced form & oxidized form, FADH2 & FAD; flavin adenine dinucleotide reduced form and oxidized form, ADP: adenosine diphosphate, ATP: adenosine triphosphate, Co Q: coenzyme Q, Cyt c: cytochrome c, UCP: uncoupling protein.

ROS are produced cellularly by enzymatic and nonenzymatic sources. Any electron-transferring protein and/or enzymatic system produce ROS as byproducts of electron transfer reactions. Although ROS are produced from NADPH oxidase, cyclooxygenases, peroxisomes, xanthine oxidase, cytochrome *P-450*, and others, mitochondria appear to produce the majority of oxidants [7, 8, 10–12, 23]. During essential oxygen-dependent ATP production in the electron transport chain (ETC), ROS is generated as a product of electron leakage from complex I and complex III where oxygen is reduced to form the superoxide radical (Figure 1) [23]. The generation of ROS in mitochondria is reported to account for ~1-2% of total oxygen consumption under reducing conditions [24]. However, the intramitochondrial concentrations of superoxide are maintained at very low steady-state levels by mitochondrial superoxide dismutase (SOD), which is present at very high concentrations [25]. 

In addition to being a main source of ROS, mitochondria are a target for oxidative damage. ROS derived from mitochondrial respiration attack mitochondrial constituents. In particular, the accumulation of somatic mutations in mitochondrial DNA (mtDNA) by oxidative stress is believed to play a key role in physiological decline associated with aging [6–8, 10–12, 26]. MtDNA is located at the mitochondrial matrix where ROS are actively generated [7, 26]. Furthermore, mtDNA lacks protective histones and has relatively low DNA repair capacity [7, 26]. ROS-induced mtDNA damage causes mtDNA mutations if mtDNA damage is not promptly repaired [16, 27]. There is a positive relationship between the increase in oxidative damage to mtDNA and the age-associated increase in mtDNA deletions and point mutations [2]. Because mtDNA encodes 13 of mitochondrial proteins in the ETC complexes [28, 29], mtDNA mutations alter the coupling of electron transport and ATP production. Finally, mtDNA mutations increase electron leakage from the ETC complexes and further damage mtDNA, as well as other important organelles [9]. Therefore, the mitochondrion is believed to be the key organelle in the cellular aging process.

## 3. Age-Associated Alterations in Cardiac Mitochondrial Function

The heart exhibits a highly aerobic metabolism due to the abundance of large mitochondria, which produce the huge amount of ATP for continuous contraction. Therefore, the relationship between aging and mitochondrial bioenergetics in cardiomyocytes has been an important research area for many years. However, the interpretation of age-associated alterations in cardiac mitochondrial function has been complicated by several factors. The heart contains two structurally similar but biochemically distinct mitochondrial populations [8, 30, 31]. Subsarcolemmal mitochondria are located beneath the plasma membrane, whereas interfibrillar mitochondria are arranged in parallel with myofibrils [8, 30, 31]. Although evidence demonstrates that 2 populations of mitochondria differ in morphology and function, the isolation procedure to evaluate age-associated alterations in cardiac mitochondrial function yields either subsarcolemmal mitochondria alone or a mixed population of both mitochondria in most reports [31–34]. In addition, cardiomyocytes with dysfunctional mitochondria might be likely to drop out via apoptosis, necrosis, and/or autophagy thus further complicating the detection of bioenergetic changes in cardiac mitochondria with aging because only relatively healthy mitochondria are obtained after the isolation procedure [8]. Compared with those observed in cardiac diseases, alterations in cardiac mitochondria observed in aging might occur heterogeneously in the whole heart and at very low level among mitochondria. Therefore, the early feature of cardiac mitochondrial alterations might be masked by individual differences.

Whether mitochondrial oxidative phosphorylation is gradually impaired with aging remains controversial [33, 35–38]. Some studies have demonstrated a decline in oxygen consumption with age [36, 38], but others have reported no change [35, 37]. Fannin et al. reported that interfibrillar mitochondria but not subsarcolemmal mitochondria obtained from aged rat hearts (24 and 28 months old) exhibited less protein production and oxidative phosphorylation rates, compared with those from adult rat heart (6 months old) [32]. This finding might explain the inconsistency in age-associated alterations in oxidative phosphorylation: the ratio of interfibrillar mitochondria to total mitochondria preparations would be expected to vary. 

Whether the activity of each ETC complex decreases with aging is also undetermined [39–46]. Among the five ETC complexes, complex I appears to be susceptible to age-associated decline in activity in the mammalian heart [39, 40, 42, 44–46], although some studies demonstrated no significant change in complex I activity with age [41, 43]. Complex I is believed to be a main source of ROS derived from mitochondrial ETC and to be responsible for the increase in mitochondrial ROS production with aging [12, 23, 42, 44, 47]. Because 7 of 13 mtDNA-encoded polypeptides in the ETC are found in complex I [28, 29], this complex is likely to be most commonly affected by aging, if the mitochondrial theory of aging proves to be true [42]. In addition, the activities of complexes III and IV, which also contain mtDNA-encoded proteins, have been reported to decrease with aging [32, 34, 39–41, 45]. Complex III is another source of ROS production in the mitochondrial ETC [12, 23, 34]. In contrast, most investigations have shown that the activity of complex II appears to be unaffected or rather enhanced by aging [40, 41, 43, 45, 46]. Since mtDNA does not encode any of the polypeptides in complex II [48], this finding further supports the mitochondrial theory of aging. In addition, protein levels in complexes III, IV, and V were reported to decrease in hearts obtained from old monkeys [38]. Gómez et al. demonstrated that formation of supercomplexes consisting of complexes I, III, and IV decreased in the aged rat heart [49]. Some investigators reported that a decrease in mitochondrial cardiolipin, which is located in the inner mitochondrial membrane and bound to cytochrome c, is closely associated with the decrease in the activity of complex III [50, 51]. However, others have reported that aging does not alter mitochondrial cardiolipin content or composition in subsarcolemmal or interfibrillar mitochondria isolated from rat hearts [44]. Complex V activity is also reported to decline with aging in the heart [52–54]; oxidative modification of *β*-polypeptides in the F1 complex of complex V may be responsible, at least in part, for this phenomenon [54]. Other types of protein modification, such as 3-nitrotyrosine, have also been found in each ETC complex [55]. Recently, an age-related decline in complexes I and V activity that correlated with increased oxidative modification has been reported in the aged mouse heart, although there was no change in the protein expression levels of them [56]. They may contribute to the decline in mitochondrial function associated with aging. 

The mitochondrial proteome has been comprehensively analyzed to clarify the effect of aging and CR on mitochondrial proteins. Chang et al. demonstrated that the effect of aging on the mitochondrial proteome in the heart appears to be slighter than that in the liver, and CR has a minor effect on these changes [57]. These results strongly suggest that posttranslational modifications of mitochondrial proteins are more important than transcriptional changes in the development of age-associated alterations in mitochondrial function and the effect of CR [58].

In conclusion, mounting evidence supports an age-associated decline in cardiac mitochondrial function, especially in the activity of complexes I, III, and IV. However, future studies are required to determine the exact mechanism by which aging impairs cardiac mitochondrial function and to further characterize differential effects of age on the subsarcolemmal and interfibrillar mitochondria populations in the heart.

## 4. Mitochondrial Oxidative Damage in the Aged Heart

As a major source of ROS production, mitochondria themselves are susceptible to oxidative damage. In fact, the accumulation of oxidative damage in mitochondria is observed in various organs of aged animals [5, 10, 11, 13, 59, 60]. ROS produced by mitochondria damages mitochondrial and nuclear DNA, lipids, and proteins. Oxidized DNA may mutate, lipid peroxidation can attenuate integrity of cellular and intracellular membrane, and oxidized proteins lose their enzymatic activity [16, 60, 61]. These events negatively affect mitochondrial and cellular function and contribute to the decline in physiological function with age. Although oxidative damage increases with age in nuclear DNA and mtDNA [27], Barja and Herrero demonstrated that levels of 8-oxo-7,8-dihydro-2′-deoxyguanosine (8-oxodG), an indicator of oxidative DNA damage, were 4-fold higher in mtDNA compared with levels in nuclear DNA in hearts obtained from 8 mammalian species with various lifespans [59]. They found an inverse correlation between 8-oxodG levels in mtDNA and maximal lifespan among the different species. Since the rate of repair for 8-oxodG is believed to be similar between nuclear DNA and mtDNA [62], the higher levels of 8-oxodG in mtDNA are likely caused, at least in part, by the chronic exposure of mtDNA to ROS due to its location in mitochondria. The repair activity for damaged mtDNA increases in the aged heart, indirectly supporting the idea that the rate of mtDNA damage increases with age in the heart [63]. 

Protein and lipid constituents in mitochondria are also susceptible to oxidative modifications. Increasing evidence demonstrates that polyunsaturated fatty acids contained in membrane lipids are vulnerable to peroxidation by ROS, and lipid peroxidation has been shown to increase in cardiac mitochondria with aging [33, 35, 64, 65]. Lipid peroxidation is a major contributor to the age-associated loss of membrane fluidity; two aldehyde lipid peroxidation products, malondialdehyde (MDA) and 4-hydroxy-2-nonenal (HNE), are primarily responsible for this phenomenon [61]. MDA and HNE rapidly react with proteins and exhibit various cytotoxic effects. In particular, HNE is highly reactive with other molecules and exerts numerous effects including inhibition of protein and DNA synthase, enzymatic inactivation, and subsequent cellular dysfunction [66–68]. HNE-modified proteins become resistant to proteolytic degradation and act as noncompetitive inhibitors of the proteasome [67]. In mitochondria, HNE is mainly detoxified by aldehyde dehydrogenase (ALDH) under physiological conditions [66]. Transgenic mice carrying an Aldh2 gene with a single nucleotide polymorphism (Aldh2*2), which impairs ALDH activity, exhibit a senescent phenotype at an early stage of life [69]. However, whether the enzymatic activity and/or the expression levels of ALDH are affected in the aged heart has not been evaluated. Measurement of protein carbonyl content is a commonly used method for assessing protein oxidation. Protein carbonyls can be formed via several mechanisms including site-specific metal-catalyzed oxidation of lysine, arginine, proline, and threonine residues; glycation reactions; and interaction of amino acid side chains with lipid peroxidation products such as MDA and HNE [60, 70]. The accumulation of oxidized proteins may play a role in the loss of physiological function with age because oxidized proteins lose catalytic activity and are prone to forming large and potentially cytotoxic protein aggregates [5, 60, 70]. Several studies have shown an age-associated increase in protein carbonyls in cardiac mitochondria and concluded that this increase may contribute, at least in part, to the decline in mitochondrial function with aging [33, 35, 39, 71].

## 5. The Effect of CR on Cardiac Oxidative Stress

CR is the only experimental intervention that has consistently shown to slow the rate of aging and increase both mean and maximal lifespan in various species [11, 13–15]. The exact mechanisms by which CR extends lifespan have not fully been evaluated, but mounting evidence demonstrates that a reduction in oxidative stress contributes, at least in part, to the antiaging effects of CR [11, 13–15, 19, 71, 72]. CR attenuates the age-associated increase in mitochondrial ROS production, lipid peroxidation, protein oxidation, and oxidative damage of mtDNA in various organs.

 Increasing evidence demonstrates that CR has pleiotropic effects on the cardiovascular system [15, 73]. Several studies have reported that CR significantly decreases oxidative damage in the aged heart [47, 71, 74–76]. The levels of 8-oxodG were lower in cardiac mitochondria obtained from CR rats than in those from ad libitum-fed controls [47, 76]. Numerous reports indicate that CR reduces oxidative damage to proteins and lipids in cardiac mitochondria. However, the exact mechanisms involved remain to be determined. In other words, how CR attenuates cardiac oxidative stress has not been established. 

Recently, sirtuin 3 (SIRT3) located in mitochondria was shown to play an essential role in enhancing antioxidant defense during CR in the liver and the brain [77, 78]. SIRT3 is a member of the sirtuin family which comprises seven proteins (SIRT1-SIRT7), whose tissue specificity, subcellular localization, enzymatic activity, and target proteins vary (Table 2) [79–81]. Sirtuins have received significant attention since the discovery that a yeast sirtuin, *silent information regulator (Sir) 2,* extends yeast lifespan. Sir2 was identified as an NAD^+^-dependent histone deacetylase. Sirtuins deacetylate histones and a wide range of transcriptional regulators and intracellular molecules, thereby controlling their activity. Although it remains controversial whether sirtuins mediate lifespan extension afforded by CR, sirtuins do regulate various aspects of the CR response, namely, glucose homeostasis, insulin secretion, fat metabolism, stress resistance, and physical activity. However, direct evidence that sirtuins play a key role in the reduction of oxidative stress in the cardiovascular system was lacking.

**Figure 2:**
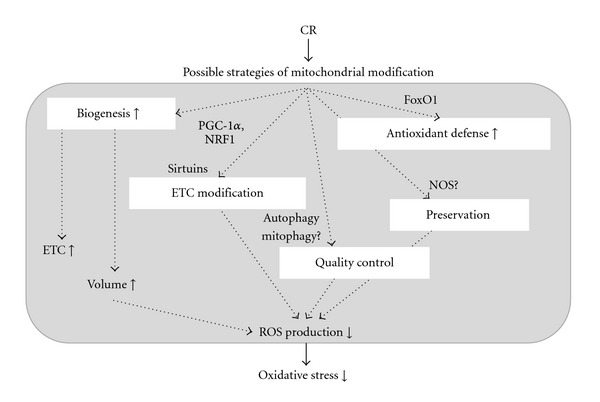
Possible mechanisms by which CR attenuates oxidative stress. CR: caloric restriction, PGC-1*α*: peroxisome proliferator-activated receptor *γ* coactivator 1*α*, NRF1: nuclear respiratory factor 1, FoxO1: forkhead transcriptional factor 1, NOS: nitric oxide synthase.

Several studies have reported that CR decreases mitochondrial ROS production in the heart, but failed to clarify the mechanism underlying this observation [47, 82–84].  Figure 2 presents possible mechanisms by which CR attenuates cardiac oxidative damage, which are verified in the latter half of this paper.

## 6. Antioxidant Defense in the CR Heart

CR might modify mitochondrial ROS production via enhanced mitochondrial biogenesis associated with amplified antioxidant mechanisms. Nisoli et al. demonstrated that 3 months of CR enhanced mitochondrial biogenesis in the murine heart and in skeletal muscle [85]. They concluded that activation of SIRT1 contributes to an increase in mitochondrial biogenesis in the CR heart by upregulating gene involved in mitochondrial biogenesis, including nuclear respiratory factor 1 and peroxisome proliferator-activated receptor *γ* coactivator 1. Activation of SIRT1 might enhance the expression of manganese SOD (MnSOD) by activating Forkhead box protein O1 as observed in cardiomyopathy hamsters treated with resveratrol [86]. However, we did not find any increase in the mitochondrial DNA and protein content or in the expression of MnSOD in middle-aged rat hearts treated with 6 months of CR, indicating that CR did not enhance mitochondrial biogenesis associated with amplified antioxidant mechanisms [84]. In this regard, Colom et al. failed to find any increase in the expression levels of MnSOD in 18-month-old rat hearts treated with 3 months of CR [82]. More recently, SIRT3 was reported to enhance the enzymatic activity of MnSOD by direct deacetylation [77]. However, it has not been determined yet whether MnSOD is the target of SIRT3 in cardiomyocytes. Judge et al. demonstrated that the activity of MnSOD was significantly reduced in subsarcolemmal mitochondria from young rat hearts treated with CR for 8 weeks, associated with a decrease in H_2_O_2_ production [83]. Therefore, the simplest explanation is that CR attenuates mitochondrial oxidative damage by suppressing mitochondrial ROS production, rather than by enhancing antioxidant defense.

## 7. Mitochondrial Function in the CR Heart

Results on the effect of CR on basal mitochondrial function in the heart are inconsistent [47, 74, 75, 82, 84, 87]. We have evaluated the enzymatic activity of the ETC, baseline mitochondrial respiration, and mitochondrial H_2_O_2_ production in the hearts of middle-aged (12-month-old) rats treated with 6 months of CR and those of middle-aged rats fed AL [84]. The only difference was that CR attenuated maximal H_2_O_2_ production in mitochondria; this was assessed by adding rotenone (complex I inhibitor) in the presence of pyruvate/malate.

Several studies demonstrated that prolonged CR improved mitochondrial function at baseline in aged hearts, but other studies failed to find any changes in mitochondria [58]. The discrepancy among studies seems to depend on the strain of rats used, the age of rats analyzed, and the duration of CR treatment. Niemann et al. demonstrated that 6 months of CR improved mitochondrial respiration and enhanced the enzymatic activity of complex I in 30-month-old rat hearts but not in 12-month-old rat hearts [75]. Our results were consistent with their findings in middle-aged rat hearts, suggesting that CR has little effect on basal mitochondrial function in the middle-aged heart, probably because mitochondrial function is maintained at a high level [84].

We speculated that the difference in mitochondrial function between AL and CR might be more remarkable under stressful conditions, such as ischemia/reperfusion. As expected, CR preserved state 3 respiration and increased the respiratory control index in the presence of pyruvate/malate in the ischemic-reperfused heart [84]. These findings suggest that the mitochondria in the CR heart are well coupled during the ischemia/reperfusion sequence. Mitochondria obtained from ischemic-reperfused CR hearts produced less H_2_O_2_ in the presence of pyruvate/malate [84], suggesting that mitochondria in the CR heart produce less ROS during early reperfusion. The preservation of mitochondrial respiration with attenuated H_2_O_2_ production in the CR heart subjected to ischemia/reperfusion strongly suggests that mitochondria in the CR heart are more resistant to ischemia/reperfusion. Suppressing mitochondrial ROS production under various stresses could attenuate the long-term accumulation of oxidative damage and might retard cardiac senescence in the CR heart [58].

## 8. Possible Mechanism of Attenuated Oxidative Damage in the CR Heart

Increasing evidence indicates that several enzymes target mitochondria under stress. Thus, it is likely that mitochondrial proteins are modified posttranscriptionally and regulated to allow the cell to adapt to various stresses. Among many forms of protein modification, we investigated the acetylation/deacetylation of mitochondrial proteins in order to clarify the effects of CR. Three members of the sirtuin family, SIRT3, SIRT4, and SIRT5, localize specifically in mitochondria and play an important role in regulating mitochondrial metabolism and function by modifying target mitochondrial proteins in various organs under metabolic stress [79–81]. In addition, we previously demonstrated that cardioprotection afforded by prolonged CR is associated with an increase in SIRT1 in the nuclear fraction [88].

We found that the levels of proteins acetylated at lysine residues increased with age in the mitochondrial fraction; furthermore, these changes were partially attenuated with CR [84]. There was no increase in the expression of SIRT3, 4, or 5 in mitochondrial fractions obtained from the CR heart. However, CR significantly increased NAD^+^-dependent deacetylase activity in total heart homogenate and the mitochondrial fraction [84]. These results suggest that CR enhances the activity of sirtuins and that activated sirtuins might be responsible for the reverse effect of CR on mitochondrial protein acetylation associated with aging.

Among the mitochondrial proteins deacetylated during CR, we focused on the following 2 mitochondrial proteins involved in the ETC: NADH-ubiquinone oxidoreductase 75 kDa subunit (NDUFS1) and cytochrome bc1 complex Rieske subunit [84]. NDUFS1 is the largest subunit of complex I and is a component of the iron-sulfur fragment of the enzyme. It faces the mitochondrial matrix, receives electrons from NADH, and passes them to the downstream iron-sulfur protein clusters. The Rieske subunit is also a component of the iron-sulfur fragment of cytochrome bc1 complex and transfers electrons to cytochrome c. Although the exact mechanism has not been clarified, we speculate that deacetylation of these proteins might stabilize electron transfer through the ETC and reduce electron leakage during ischemia/reperfusion sequence. As described previously, many mitochondrial proteins in the ETC are targets for oxidative modifications under various stresses. In contrast, it is plausible that some types of posttranscriptional modification play a protective role against oxidative modification under stress [58]. 

## 9. Reduction in Mitochondrial Burden during CR

Among various substrates that the heart can utilize, free fatty acids are believed to be the major substrate for the adult heart under physiological conditions. In contrast, utilization of glucose in the heart predominantly increases under pathophysiological conditions such as ischemia/reperfusion and pressure overload. The efficiency of ATP production per one molecule of substrate is better when fatty acids are utilized, but mitochondria require high levels of oxygen for *β*-oxidation during fatty acid metabolism. Finally, both glucose-derived and fatty acid-derived acetyl CoA enter the TCA cycle. Therefore, the question arises whether the preference of energy substrate is altered in the CR heart. 

 Sung et al. evaluated the effect of short-term CR (for 5 weeks) on myocardial metabolism [89]. They found that there were no differences in rates of palmitate oxidation and glycolysis during aerobic perfusion between the CR and the AL hearts. However, glucose oxidation was increased by 175% in the CR heart, compared to the AL heart. Consistent with this finding, glucose-derived acetyl CoA production in the TCA cycle was increased in the CR heart. A high rate of glucose oxidation in the CR heart might contribute to improve myocardial energetics under stressful conditions. However, there is no data regarding the effect of long-term CR on myocardial metabolism and this issue remains to be clarified. 

 Experimental studies demonstrate that animals subjected to CR exhibit a decrease in systemic blood pressure and heart rate, compared with controls fed AL [90, 91]. A clinical observation in which individuals who had been on CR diet for an average of 6 years were compared with age-matched healthy individuals on typical American diet indicated a change in blood pressure similar to that observed in animal experiments [92]. Thus, the decrease in cardiovascular burden during CR might contribute, at least in part, to the antiaging effect of CR on cardiovascular senescence. Recent studies demonstrated that SIRT1 and endothelial nitric oxide synthase (eNOS) colocalize in endothelial cells and that SIRT1 deacetylates eNOS, stimulating eNOS activity, and increasing NO production in endothelial cells [15, 93, 94]. Thus, it is plausible that CR activates SIRT1 and improves NO bioavailability in the cardiovascular system, resulting in a decrease in blood pressure during CR [95]. Reduction of mitochondrial work in the heart by pharmacological intervention has potential for mimicking the effect of long-term CR. In this regard, either pharmacological inhibition of renin-angiotensin system throughout the lifespan or genetic disruption of the angiotensin type 1 receptor gene, which led to a decrease in blood pressure, was reported to promote longevity and retard cardiovascular senescence in rodents [96, 97]. 

## 10. Implications for Human Aging and Age-Associated Cardiovascular Diseases

Although it is well accepted that oxidative stress is involved in the aging process and the antiaging effect of CR is closely related to a reduction in ROS-induced cellular damage, there is very little data that demonstrates these relationships in humans or lager animals. Therefore, the translatability to human aging for short-lived small species is always a debating issue. A clear distinction should be drawn between the observation that CR appears to work in humans and a recommendation that individuals embark on its practice. A lot of issues have remained to be understood about the effect of CR on humans.

 An increased baseline level of oxidative damage to DNA is reported to be associated with age [98] and several age-related diseases including cardiovascular diseases [99]. Higher levels of protein carbonyls are observed with increased age in healthy human subjects [100]. In contrast, a recent investigation from comprehensive assessment of the long-term effect of reducing intake of energy (CALERIE) demonstrated that DNA damage was reduced from baseline after 6 months in individuals assigned to CR, but not in controls [101]. Although the effects of CR on lifespan in nonhuman primates were inconsistent [102, 103], preferable outcomes on age-associated oxidative damage by CR could be observed in nonhuman primates [104]. These results suggest that the oxidative stress theory of aging would apply to humans, at least in part.

Clearly, the use of CR mimetics is much easier to incorporate into clinical practice than lifelong CR. The role of sirtuins on lifespan extension by CR remains controversial, but there is the fact that sirtuins regulate various aspects of the CR response. In addition, SIRT1 plays an important role in cardiac adaptive response to various stresses such as ischemia/reperfusion [105] and activation of SIRT1 confers antioxidative and anti-inflammatory effects in the vasculature, resulting in attenuated vascular senescence [106]. Thus, activators of sirtuins are potentially useful for managing age-associated cardiovascular diseases. Our finding further suggests targeted modification of specific mitochondrial proteins by relevant sirtuin-activating compounds is a promising approach for controlling cardiovascular senescence. We found that low-dose resveratrol mimics the effect of CR on deacetylation of specific mitochondrial proteins belonging to the ETC [84]. Resveratrol is reported to be a potentially cardioprotective compound, but not a specific sirtuin activator [107, 108]. Thus, future studies should focus on discovering other sirtuin-activating compounds that deacetylate specific mitochondrial proteins with high specificity and efficiency. The distinct role of sirtuin member should be clarified to understand the mechanism by which CR modifies mitochondrial bioenergetics and cellular oxidative stress in the cardiovascular system. At the present time, the easy use of sirtuin-activating compounds in clinical settings should be avoided.

## Figures and Tables

**Table 1:** Major biological theories of aging.

(A) The programmed theory	
(1) Programmed theory Telomere shortening theory	Aging is the result of a sequential switching of certain genes. Telomere plays a role in the genomic instability with aging.
(2) Neuroendocrine theory	Biological clocks act through the neurohumoral system to control the pace of aging.
(3) Immunological theory	The immune system is programmed to decline, which leads to an increased vulnerability to acute and chronic inflammation, resulting in aging and death.

(B) The damage or error theory (Nonprogrammed theory)	

(1) Wear and tear theory	Cells and tissues have vital parts that wear out, that leads to aging.
(2) Rate of living theory Metabolic theory	The greater a rate of basal oxygen metabolism, the shorter its lifespan.
(3) Cross-linking theory Glycation theory	The accumulation of modified constituents, such as cross-linked and glycated proteins, damages cells and tissues, resulting in aging.
(4) Free radical theory Oxidative stress theory Mitochondrial theory	Free radicals and reactive oxygen species (ROS) cause cellular damage and the accumulation of oxidative damage leads to aging. Mitochondria are a main source of ROS and also a target of ROS.
(5) Somatic DNA damage theory	DNA damages occur continuously in living cells. Most of these damages are repaired, whereas some accumulate, resulting in cellular dysfunction and aging. In particular, damages to mitochondrial DNA lead to mitochondrial dysfunction.

**Table 2:** Seven members of sirtuin family.

Sirtuin	Class	Cellular localization	Enzymatic activity	Target molecules
SIRT1	I	Nucleus, Cytosol, (Mitochondria)	Deacetylase	Histone H3, H4, p53, nuclear factor *κ*B (NF *κ*B), peroxisome proliferator-activated receptor-*γ* co-activator 1 *α* (PGC1*α*), forkhead box O transcriptional factors (FoxO) 1 & 3, Notch, hypoxia-inducible factor 1*α* (HIF1*α*), liver X receptor (LXR), farnesoid X receptor (FXR), sterol-response element-binding protein 1c (SREBP1c), p300, endothelial nitric oxide synthase (eNOS), peroxisome proliferator-activated receptor (PPAR) *γ*, CREB-regulated transcription co-activator 2 (CRTC2), and so forth
SIRT2	I	Cytosol, (Nucleus)	Deacetylase	*α*-tubulin, phosphoenolpyruvate carboxykinase (PEPCK), FoxO1, partitioning defective 3 homologue (PAR3)
SIRT3	I	Mitochondria	Deacetylase	Long-chain acyl CoA dehydrogenase (LCAD), 3-hydroxy-3-methylglutaryl CoA synthase 2 (HMGCS2), glutamate dehydrogenase (GDH), NADH dehydrogenase ubiquinone 1 subcomplex 9 (NDUFA9), superoxide dismutase 2 (SOD2), isocitrate dehydrogenase 2 (IDH2), cyclophilin D (CypD), acetyl-CoA synthetase 2 (AceCS2), LKB1, and so forth
SIRT4	II	Mitochondria	ADP-ribosyltransferase	GDH
SIRT5	III	Mitochondria	Deacetylase Demalonylase Desuccinylase	Carbamoyl phosphate synthetase 1 (CPS1)
SIRT6	IV	Nucleus	Deacetylase ADP-ribosyltransferase	Histone H3K9, H3K56
SIRT7	IV	Nucleolus	Unknown	Histone H3K18, p53, RNA polymerase I
